# Injectional anthrax: the inflammatory response

**DOI:** 10.1186/cc11110

**Published:** 2012-03-20

**Authors:** M Booth, A Hart, L Donaldson

**Affiliations:** 1 Royal Infirmary, Glasgow, UK

## Introduction

From December 2009 to July 2010 there were 47 cases of anthrax amongst injecting drug users in Scotland with 13 fatalities. The majority presented as severe soft tissue infection following i.v. injection or muscle popping as described by Ringertz and colleagues [1]. At first they were diagnosed as necrotising fasciitis (NF) until the diagnosis of anthrax was made. With experience they appeared to have a milder inflammatory response to their infection compared to other soft tissue infections such as NF. To investigate this the anthrax group was compared to a cohort of confirmed NF cases.

## Methods

Patients admitted to the ICU with NF or injectional anthrax from 1 January 2008 to 30 June 2011 were identified. The white blood count (WBC) and C-reactive protein (CRP) at presentation were recorded. Demographic data (sex, age, ICU and hospital LOS, APACHE II score, predicted and actual hospital mortality and drug-injecting history) were retrieved. All data were collected prospectively for routine ICU management.

## Results

There were six patients with injectional anthrax and 16 with NF. The results are presented in Table 1. There was a marked difference in the inflammatory response between the two groups with the CRP being highly statistically significant. The anthrax group was also younger (35.5 vs. 43.2) with a lower severity of illness, lower predicted mortality (18.6% vs. 31.7%) but much higher actual mortality.

**Table 1:** 

	Anthrax	NF	
Number	6	16	
APACHE II score	12.2	19.4	*P *< 0.05
Died (%)	66.6	18.8	*P *< 0.05
WBC	11.6	16.0	NS
CRP	71.2	287.3	*P *< 0.001

## Conclusion

Anthrax releases three factors: lethal factor (LF), edema factor (EF) and protective antigen (PA). PA and LF form lethal toxin which kills macrophages and inhibits B-cell and T-cell function so minimising the immune response to anthrax. This is reflected in the inappropriately low CRP levels at presentation. Severe soft tissue infection in an injecting drug user associated with subjectively poor inflammatory response should raise the possibility of anthrax infection.
